# Magnitude and correlates of virological failure among adult HIV patients receiving PI based second line ART regimens in north western Tanzania; a case control study

**DOI:** 10.1186/s12879-019-3852-3

**Published:** 2019-03-07

**Authors:** Daniel W. Gunda, Semvua B. Kilonzo, Tarcisius Mtaki, Desderius M. Bernard, Samwel E. Kalluvya, Elichilia R. Shao

**Affiliations:** 1 Department of Medicine, Bugando Medical centre, 1370 Mwanza, Tanzania; 2 Department of medicine, Weill Bugando School of Medicine, 1464 Mwanza, Tanzania; 3 Department of Medicine, Kilimanjaro Christian Medical University College, 2240 Moshi, Tanzania

**Keywords:** Second line ART, Virological failure on second line ART, Northwestern Tanzania

## Abstract

**Background:**

With a growing access to free ART, switching of ART to second line regimen has also become common following failure of first line ART regimens. Patients failing on first line ART regimens have been shown to stand a high risk of failing on subsequent second line ART regimens. The magnitude of those who are failing virologicaly on second line ART is not documented in our setting. This study was designed to assess the magnitude and correlates of second line ART treatment failure.

**Methods:**

A retrospective analysis of patients on second line ART for at least **1** year was done at Bugando care and treatment center. Information on demographic, clinical and laboratory data were collected and analyzed using STATA 11. The proportion of patients with Virological failure was calculated and potential correlates of virological failure were determined by logistic regression model.

**Results:**

In total 197 patients on second line ART were included in this study and 24 (12.18%) of them met criteria for virological failure. The odds of having virological failure on second line ART were independently associated with age of less than 30 years (AOR = 12.5, *p* = 0.001), being on first line for less than 3 years (AOR = 6.1, *p* = 0.002) and CD4 at switch to second line ART of less than 200cells/μl (AOR = 16.3, *p* < 0.001).

**Conclusion:**

Virological failure among patients on second line ART is common. Predictors of virological failure in this study could assist in planning for strategies to improve the outcome of this subgroup of patients including close clinical follow up of patients at risk, a continued adherence intensification and a targeted resistance testing before switching to second line ART.

## Background

The patients experiencing first-line antiretroviral therapy (ART) treatment failure have been shown to stand a higher risk of failing on subsequent second line regimens due to side effects and complex regimens which could lead into selection of resistant strains [1]. These patients could potentially transmit the resistant strains which are a big challenge in resource limited settings like ours where resistance testing is not practicable [2, 3]. A better strategy for HIV control would in this manner necessitate a durable and potent ART regimen. Furthermore this also requires early detection and appropriate revision of the failing ART regimens and timely switch to potent second line ART regimens [4, 5].

Switching to potent second line ART regimens has been shown to have a number of benefits including improving virological containment and immune response [6–8], reduced probable drug resistance [9] and reduced mortality [10]. Even with these advantages still a proportion of patients have been reported elsewhere to experience treatment failure while on second line ART regimen with varying magnitudes of this problem [11–13]. Virological failure on first line ART has been associated with poor adherence and its persistent may seriously limit the durability of second line ART regimen [14]. Second line ART regiments are expensive and usually more complex and are potentially associated with higher levels of side effects which may complicate non adherence and increase the risk of resistance and virological failure [15]. Other reported predictors of virological failure include WHO clinical stage 3&4 and severe immune suppression at switch to second line ART [16], and falling CD4 counts on receipt of second line ART has been shown to have a strong reciprocal relation to virological improvement [17] among others.

The literature on the extent of this problem in our setting is still scarce. This study therefore was designed to determine the prevalence and assess the risk factors of treatment failure among adult HIV patients who are receiving protease inhibitor (PI) based second line ART regimen in northwestern Tanzania. This information is important in devising strategies to optimize the outcome of this subgroup of patients but we also believe it will significantly add to the existing body of knowledge on virological performance of PI based second line ART regimen in resource limited settings like ours.

## Methods

This was a case control study which was done during elective research period from September 2017 to May 2018 at Bugando medical center (BMC) HIV Care and treatment center (CTC). BMC is a tertiary level and a university teaching hospital located in the North Western part of Tanzania. Currently the hospital has a capacity of 1000 beds, and serving about 16 million people. As one of its core services BMC also runs HIV activities with a referral CTC department. The center started a way back 2004, and it currently serves a total of more than 14,000 clients. Core activities at CTC include provision of ART, follow up and treatment monitoring. ART drugs are provided for free of charge and about 10,000 patients are currently on ART. In our setting first line ART are basically Non Nucleoside/ Nucleotide Reverse Transcriptase Inhibitors (NNRTI) based regimens; whereas patients failing on first line ART regimens are usually switched to Protease Inhibitor (PI) based second line ART regimen. Around 500 patients are currently receiving PI based second line ART regimen. ART treatment monitoring is routinely done using clinical and immunological parameters where CD4 counts are checked 6 monthly since viral loads (VL) are still expensive for routine use. Targeted VL and adherence intensification are usually done before switching to second line ART is done. Patient with VL of more than 1000copies/ml usually get a repeat testing after 3 months of adherence intensification and those with log drop of less than 0.5 are classified as failing virologicaly. Our current cohort of patients on PI based second line ART had a regular VL test 6 monthly. Those found with VL of more than 1000copies/μl had adherence intensification and a conclusion of virological failure was reached if a subsequent VL was more than 1000copies/μl. The adherence was assessed by a combination of patients’ report and pill count as done previously [18] where adherence rate of less than 95% denotes poor adherence. In our setting as it is also true for most of the resource limited settings no resistance testing is done prior switching to second line ART regimen to decrease possibilities of cross resistance.

The study involved all adult HIV positive patients who are currently receiving PI based second line ART regimens at Bugando CTC in Mwanza. A minimum sample size of 178 was estimated from Leslie Kish formula (1965) for cross sectional studies assuming 13.35% of patients on second line ART will experience virological failure as found previously [19] with an allowable error of 0.05 at 95%CI. The sample was derived from a list of all patients who were started on second line ART regimen at BMC. A CTC data base was reviewed to identify all patients who were on PI based second line ART and registration numbers were used to trace the files. The files were then numbered to ease sampling. A systematic sampling was done to get the required sample size using a skip interval of 2 where the starting point was randomly selected as done previously [20]. Files of patients who were on PI based second line ART for less than a year and those who were transferred out were not included and the next file was selected in its place. The data regarding age, sex, date of HIV diagnosis, WHO clinical stage, CD4 at initiation of first line ART, year of ART initiation and regimen, VL at switch to second line, time in months on first line, duration in months on second line ART and ART regimen, adherence status, most recent VL, and CD4 counts at switch to second line were extracted.

Data were computerized using Epi data version 3.1 and STATA version 11 (Stata Corp LP, college station, TX) was used for data analysis. Continuous variables were expressed as medians with interquartile range (IQR) while categorical variables were expressed as proportions with percentages. The treatment outcome in this study was defined as development of virological failure as used previously where patients who had a second VL of more than 1000copies/μl while receiving PI based second line ART regimen for at least a year were coded as failing virologicaly [12]. The proportion of patients with virological failure on second line ART was then calculated and expressed as percentage and the effect of different risk factors on the odds of developing virological failure was investigated. The Odds ratio with 95% Confidence Interval (CI) was calculated using univariate analysis followed by multivariate analysis model to assess the association of different variables to the outcome of interest. In all our analysis factors were said to be statistically significant when the *p* value was less than 0.05.

### Ethical clearance

The permission to conduct and publish the results from this study was sought from the catholic University of Health and allied Sciences/ Bugando Medical Center joint ethical committee. The patient’s files were handled by the researchers alone and the patients’ identifiers including names and registration numbers were not included in the analysis to further maintain confidentiality.

## Results

**Fig. 1:**
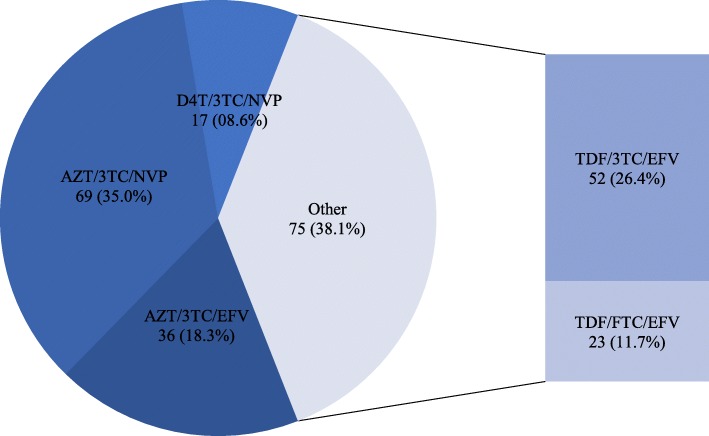
Distribution of first line ART regimen among 197 adult HIV positive participants. AZT: Zidovudine, 3TC: Lamivudine, D4T: Stavudine, EFV: Efavirenz, FTC, Emtricitabine, NVP: Nevirapine, TDF: Tenofovir

**Fig. 2:**
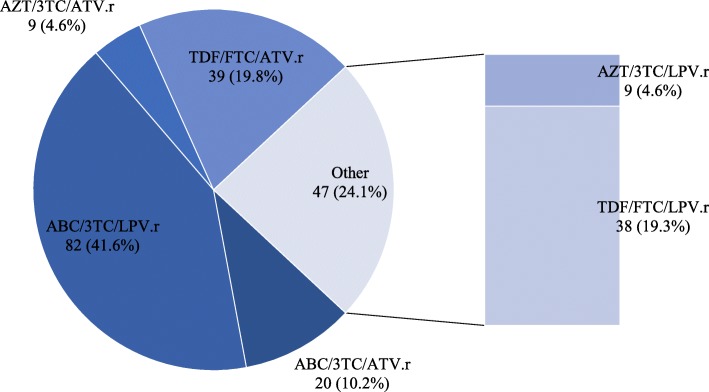
Distribution of PI based second line ART regimen among 197 adult HIV positive participants. ABC: Abacavir, AZT: Zidovudine, ATV.r: ritonavir boosted Atazanavir, 3TC: Lamivudine, FTC, Emtricitabine, LPV.r: ritonavir boosted Lopinavir, TDF: Tenofovir

In total 197 patients were enrolled in this study. About 3 quarters, 146 (74.11%) of the study participants were female patients and almost a third of participants, 64 (32.65%) had lost their spouse. At enrollment to CTC most patients, 114 (57.87%) had WHO clinical stage 3 & 4 AIDS defining illnesses and 57 (28.93%) had severe immune suppression. With a median time on second line ART of 14 [13–18] months, 26 (13.20%) participants had an associated severe immune suppression at initiation of second line ART and most patients, 135 (68.53%) were coded as having WHO stage 3&4. Though most patients had good adherence, still 25 (12.69%) had poor adherence to medication as summarized in Table 1. Overall Zidovudine based regimens were the most common, 105 (53.3%) first line ART regimen and most patients, 1129 (65.5%) were subsequently switched to ritonavir boosted Lopinavir based regimen as shown in Figs. 1 and 2 respectively.

**Table 1:** General Characteristics of 197 participants on PI based second line ART regimen

Variable	Frequency	Percent or median (IQR)
Age (years)	197	48 [41–54]
Sex		
Male	51	25.89
Female	146	74.11
Marital status		
Married	56	28.43
Divorce	36	18.27
Widow	64	32.65
single	40	20.30
WHO stage at ART1		
3&4	114	57.87
1&2	83	42.13
CD4 at ART1		
Median (cells/μl)	197	295.5 [179–406]
< 200cells/μl	57	28.93
≥ 200 cells/μl	140	71.07
WHO stage at ART2		
3&4	135	68.53
1&2	62	31.47
CD4 at ART2		
Median (cells/μl)	197	484 [341–700]
< 200cells/μl	26	13.20
≥ 200cells/μl	171	86.80
First line ART months	197	113 [46–133]
Second line ART months	197	14 [13–18]
Adherence < 95%		
Yes	25	12.69
No	172	87.31
Viral load (copies/μl)		
≥ 1000	24	12.18
< 1000	173	87.82

*CD4* Cluster of differentiation 4, *CD4 at ART1* denotes CD4s at initiation of first line ART, *CD4 at ART2* CD4 counts at switch to second line ART, *IQR* Interquatile range, *WHOs* World health Organization clinical stage, *WHO stage at ART1* denotes WHO clinical stage at initiation of first line ART, *WHO stage at ART2* Denotes WHO clinical stage at switch to second line ART

A total of 24 (12.18%) participants were found to meet criteria for virological failure in this study (Table 1). The odds of having virological failure on PI based second line ART in this study were independently associated with age of less than 30 years (AOR = 12.4, *p* = 0.001), being on first line ART regimen for less 3 years (AOR = 7.6, *p* = 0.003) and CD4 counts of less than 200cells/μl at switch to second line ART (AOR = 16.3, *p* < 0.001). Adherence level of less than 95% had a significant association with virological failure on univariate analysis alone (OR = 3.4, *p* = 0.028) while the difference in distribution of other variables was not different statistically including the type of ART regimens and time on second line ART as summarized in Table 2.

**Table 2:** Univariate and multivariate analysis for factors associated with virological failure among 197 adult HIV patients on PI based second line ART

Variables	Virological failure		Unadjusted		Adjusted	
	No (*n* %)	Yes (*n* %)	OR (95%CI)	*P*-value	OR (95%CI)	*P*-value
Sex						
Male	042 (24.28)	09 (37.50)				
Female	131 (75.72)	15 (62.50)	1.9 (0.7–4.5)	0.171		
Age						
< 30 years	012 (06.94)	07 (29.17)				
≥ 30 years	161 (93.06)	17 (70.83)	5.5 (1.9–15.9)	0.002	12.4 (2.7–56.1)	0.001
Marital state						
Married	46 (26.59)	10 (41.67)	1.9 (0.8–4.7)	0.130	–	–
Divorced	33 (19.08)	03 (12.50)	0.6 (0.2–2.2)	0.439	–	–
Widow	59 (34.30)	05 (20.83)	0.5 (0.3–1.4)	0.194	–	–
Single	34 (19.65)	06 (25.00)	1.3 (0.5–3.6)	0.543	–	–
WHO stage at ART1						
3&4	101 (58.38)	13 (54.17)				
1&2	072 (41.62)	11 (45.83)	0.8 (0.3–2.0)	0.695	–	–
ART1months						
≤ 36 months	015 (08.67)	08 (33.33)				
> 36 months	158 (91.33)	16 (66.67)	5.3 (1.9–14.3)	0.001	7.6 (1.9–29.0)	0.003
CD4 at ART2						
< 200cells/μl	035 (20.23)	18 (75.00)				
≥ 200 cells/μl	138 (79.77)	06 (25.00)	11.8 (4.3–32)	< 0.001	16.3 (5.-57.9)	< 0.001
WHO stage at ART2						
3&4	121 (69.94)	14 (58.33)				
1&2	052 (30.06)	10 (41.67)	0.6 (0.3–1.4)	0.255	–	–
ART2 months	009 [05–11]	6 [4.5–9.5]	0.9 (0.7–1.0)	0.065	–	–
Adherence < 95%						
Yes	017 (09.83)	08 (33.33)				
No	156 (90.17)	16 (66.67)	4.5 (2.3–12.3)	0.002	3.4 (0.9–12.6)	0.062
ART1rgm						
AZT/3TC/EFV	32 (18.50)	04 (16.67)	0.9 (0.3–2.8)	0.828	–	–
AZT/3TC/NVP	66 (38.15)	03 (12.50)	0.2 (0.1–0.8)	0.022	–	–
D4T/3TC/NVP	15 (08.67)	02 (08.33)	0 .9 (0.2–4.4)	0.956	–	–
TDF/3TC/EFV	60 (34.68)	15 (62.50)	3.1 (2.1–7.6)	0.011	0.5 (0.2–1.9)	0.363
ART2rgm						
ABC/3TC/LPV.r	86 (49.71)	16 (66.67)	2.1 (1.8–4.9)	0.0125	2.1 (0.5–7.8)	0.272
AZT/3TC/ATV.r	07 (04.05)	02 (08.33)	2.1 (0.4–11.)	0.357	–	–
AZT/3TC/LPV.r	07 (04.05)	02 (08.33)	2.1 (0.4–11)	0.357	–	–
TDF/FTC/ATV.r	37 (21.39)	01 (04.70)	0.2 (0.2–1.2)	0.077	–	–
TDF/FTC/LPV.r	36 (20.81)	03 (12.50)	0.4 (0.2–1.9)	0.346	–	–

*ABC* Abacavir, *ART1rgm* first line ART regimen, *ART2rgm* second line ART regimen, *AZT* Zidovudine, *ATV.r* ritonavir boosted Atazanavir, *3TC* Lamivudine, *CD4* Cluster of differentiation 4, *CD4 at ART2* CD4 counts at switch to second line ART, *CI* Confidence interval, *D4T* Stavudine, *EFV* Efavirenz, *FTC* Emtricitabine, *IQR* Interquatile range, *LPV.r* ritonavir boosted Lopinavir, *n* number, *NVP* Nevirapine, *rgm* regimen, *TDF* Tenofovir, *WHO stage at ART1* Denotes World health Organization clinical stage at initiation of first line ART, *WHO stage at ART2* Denotes WHO clinical stage at switch to second line ART

## Discussion

The objective of this study was to determine the prevalence and risk factors of virological failure among adult HIV patients receiving PI based second ART in northwestern Tanzania. The 12.18% rate of virological failure on PI based second line ART in this study is similar to prevalence rates of 13.35% reported earlier in 2012 from South Africa [19] and 13.9% reported in 2014 by Sigaloff among sub-Saharan patients [21]. Another comparatively higher rate of virological failure of 17.7% was reported in India among patients who were on second line ART beyond 1 year [12] and in South Africa where it was shown that about 23% of patients who failed on first line ART experienced virological failure after 1 year of treatment with second line ART [11]. A much higher rate of virological failure of 37% was also recently reported from Myanmar Asia among patient who were on second line ART [22].

Though the rate of virological failure in the index study is comparatively lower than findings from elsewhere, in general all these findings suggest that virological failure is a common phenomenon with a potential threat to second line long term outcome. One systematic review in resource limited setting indicated that virological failure is a common encounter among HIV patients on second line ARTs which tend to increase in magnitude with time on ART suggesting a possible accumulation of resistant strains as a cause of this treatment failure [23].

Several factors were investigated for possible independent association with virological failure in the index study. In our study we found that patients who experienced treatment failure after being on first line ART for less than 36 months were more likely to fail on subsequent PI based second line ART. A similar observation was reported by Boerma in 2017 indicating that of those patients who failed on first line ART regimen less than 24 months were also more likely to fail on second line ART [24]. This could be because of possible cross resistance mutations in this subgroup of patients as suggested previously [25, 26]. In Mozambique it was shown that resistance mutations were a common cause of virological failure on first line ARTs, the prevalence of which kept on increasing with time on ART [27]. This suggests that in settings like ours where switching to second line ART is not routinely guided by resistance testing, a possible selection and accumulation of drug resistant strains could possibly lead into early failure of second line ART regimen [26, 28], though the time on second line ART was not statistically different in our study between those failing virologicaly and their successful counter parts (6 months vs. 9 months, *p* = 0.065).

Additionally in this study young patients were found to have increased risk of virological failure especially among those who were younger than 30 years. Onoya and colleagues had similar observation indicating that patients who were younger than 40 years were more likely to fail on second line ART [29]. Similar observations were also reported by Boerma et al. in a multicenter analysis of second line ARTs among pediatric patients where adolescents (10-18 years) were found to have an increased risk of failing on second line ART [24]. Though poor adherence to ART medications did not show any independent association with virological failure in this study, previous studies had indicated that poor adherence is associated with virological failure and is more common among younger patients as compared to older patients [30, 31].

Poor adherence would limit virological control and a loss of immunological and clinical improvement [15]. In a recent study by Tsegaye et al. in 2016 it was indicated that patients with virological failure on second line ART regimen independently had severe immune suppression with CD4 counts of less than 100cells/μl and WHO clinical stage 3&4 AIDS defining illness at switch to second line ART regimen [16]. This report is in agreement with our finding where we also observed that patients with virological failure were about 16 times more likely to have an associated severe immune suppression at switch to PI based second line ART with CD4 counts of less than 200cells/μl as compared to their virologicaly successful counterparts though the difference in clinical deterioration was not statistically different between the two groups.

This study is potentially liable to a number of limitations. Being a single center study its results may not be generalizable. It was a retrospective study from which some information could not be retrieved including assessment of attendant opportunistic conditions. However even with these limitations this is probably the first study to evaluate virological performance of PI based second line ART without resistance guidance in Tanzania and thus we think the findings from this study are valuable and will serve as a potential foundation for further studies to maximize the outcome of HIV patients in this part of resource limited setting which is currently serving as CTC referral facility for the northwestern Tanzania. A prospective analysis of patients with probably resistance testing component might give us a better insight of this subgroup of patients.

## Conclusion

In conclusion these findings are clinically important in our setting suggesting that virological failure on present PI base second line ART is a prevailing problem though its size is relatively small in this study as compared to most other studies. Patients failing on second line ART have increased risk of mortality as shown in previous studies [32, 33]. Since third line ART are expensive and are not currently in use in our setting early identification and closer clinical follow up of patients at risk of failing on second line ART would improve their outcome. Patient at risk of virological failure on second line ART include those who failed shortly on first line ART regimen, those who were younger than 30 years. Non improving CD4 counts at switch to second line ART is also a potential marker of a subsequent virological failure in this subgroup of patients. We believe that apart from current continued adherence intensification, a targeted resistance testing might serve as a potential strategy in further improving the outcome of this subgroup of patients in our setting where third line options are still unavailable.

## Abbreviations

3TC: Lamivudine
ABC: Abacavir
AIDS: acquired immune deficiency syndrome
ART: antiretroviral therapy
ATV.r: ritonavir boosted Atazanavir
AZT: Zidovudine
BMC: Bugando medical center
CD4: cluster of differentiation 4
CI: confidence interval
CTC: care and treatment Center
CUHAS: Catholic University of Health and Allied Sciences
D4T: Stavudine
EFV: Efavirenz
FTC: Emtricitabine
IQR: Interquatile range
LPV.r: ritonavir boosted Lopinavir
NVP: Nevirapine
OIs: opportunistic infections
OR: odds ratio
PI: protease inhibitor
TDF: Tenofovir
VL: viral load
WHO: World Health Organization
